# Effect of In utero Exposure to Air Pollution on Adulthood Hospitalizations

**DOI:** 10.1007/s11524-023-00803-1

**Published:** 2023-12-08

**Authors:** Nicolau Martin-Bassols, Sonja C. de New, Michael A. Shields, David W. Johnston

**Affiliations:** 1 Centre for Health Economics, Monash Business School, 900 Dandenong Rd, Caulfield East, VIC 3145 Australia; 2 ARC Life Course Centre, Brisbane, Australia; 3 Institute of Labour Economics, Bonn, Germany

**Keywords:** Respiratory health, Great London Smog, In utero, Event study

## Abstract

Empirical analyses have demonstrated that individuals exposed to severe air pollution in utero have worse health outcomes during childhood. However, there is little evidence on the long-term health impacts of air pollution exposure. The objective of this paper is to estimate the effect of in utero exposure to the Great London Smog of 1952 (GLS) on five health outcomes identified through a scoping review to be those most likely affected: respiratory, circulatory, neoplasms, mental health, and nervous system conditions. We use the GLS, an extreme air pollution event in December 1952, as a quasi-natural experiment to estimate the effect of exposure to air pollution in utero on adulthood health. Data from the UK Biobank is analysed for a cohort of participants born from December 1952 to July 1956. Differences in health outcomes between adults exposed and not exposed to the GLS due to their birth dates, born inside and outside London, were explored. Our primary focus is hospitalization events between 1997 and 2020 (corresponding to ages 40 to 69), as recorded in linked administrative data from the National Health Service (NHS). Specifically, the five primary outcomes are binary variables indicating that the individual had at least one hospitalization where the main cause of hospitalization is related to respiratory, circulatory, neoplasms, mental health, or nervous system conditions. The analytical sample comprised 36,281 individuals. A positive effect on adulthood hospitalizations due to respiratory conditions was observed. If exposed to the GLS in utero, the probability of at least one respiratory health-related hospitalization between 1997 and 2020 increased by 2.58 percentage points (95% CI 0.08, 4.30, *p* = 0.03), a 23% increase relative to the sample mean. Small effects were found for all other outcomes, suggesting that these conditions were not affected by the GLS. We do not find heterogeneous effects by sex or childhood socioeconomic status. This study found that a 5-day pollution exposure event while in utero significantly increased respiratory-related hospitalizations at ages 40 to 69 but had no impact on hospitalizations due to circulatory, neoplasms, mental health, and nervous system conditions.

## Introduction

It has been established that severe air pollution exposure causes health problems in the immediate months and years following exposure [1]. However, there is little causal evidence on how severe air pollution exposure affects health decades afterwards [2–5]. Of particular interest are the long-term effects of sudden pollution exposure in utero, which may result from wildfires and industrial accidents [6–11]. The ‘foetal origin hypothesis’ [12–14] argues that the most critical period of human development is the gestational phase and that disruptions during this time can have long-lasting effects.

Due to the complex nature of exposure to pollution in utero, it is currently unclear which dimensions of health are impacted in the long term. Most existing studies have concentrated on conditions related to the respiratory, circulatory, neoplasms, mental health, and nervous systems (see review in Appendix 1). However, studies have typically concentrated on only one health outcome and have used different data types and methodological designs. Therefore, it remains unclear which outcomes are impacted by exposure to air pollution during foetal development and how the degree of impact differs between them.

Furthermore, despite the substantial body of evidence, the demonstrated links between in utero exposure and long-term health outcomes are primarily correlational [14–17]. A critical identification problem is that air pollution is strongly associated with other health risk factors [18]. As such, it is uncertain whether correlations between in utero exposure and adulthood health reflect causal relationships or confounding factors. This paper uses the Great London Smog (GLS) event as a quasi-natural experiment to investigate the long-term effect of exposure to air pollution in utero.

The GLS was a severe air pollution event between the fifth and the ninth of December 1952, caused by an unusual combination of a high-pressure weather system and extreme industrial pollution. Each day of the GLS, 1000 tonnes of smoke particles, 2000 tonnes of carbon dioxide, 140 tonnes of hydrochloric acid, 14 tonnes of fluorine compounds, and 370 tonnes of sulphur dioxide were emitted [19]. Studies suggest pollution levels were similarly severe across London’s high and low socioeconomic areas [20]. There are only a few studies of GLS impacts. Most have concentrated on the short-term mortality effects, and it is estimated that it caused 4000 deaths within a week and 12,000 deaths within a year [19, 21]. In terms of medium- and long-term impacts, it is estimated that in utero exposure caused an increase in childhood asthma [7] and adulthood respiratory conditions [8] and that infant exposure caused an increase in adulthood neoplasms [9]. This study contributes to this small literature by providing evidence on the effect of the GLS on hospitalizations due to respiratory, circulatory, neoplasms, mental health, and nervous system conditions. Methodologically, a difference-in-difference regression approach was applied to a large dataset of older people. Additional explorations include stratifying the data by sex, socioeconomic status, and gestational timing of exposure.

## Material and Methods

### Study Design

This is a cohort study using data from the UK Biobank and the Hospital Episode Statistics (HES) and Admitted Patient Care (APC) databases. It aims to estimate the effect of in utero exposure to air pollution on long-term respiratory, circulatory, neoplasms, mental health, and nervous system conditions. Using a difference-in-differences methodology, we compared the difference in outcomes between individuals born in London who were exposed to the GLS (in utero during) and not exposed to the GLS (born afterwards), with the difference in outcomes between individuals born outside of London during and after the GLS.

### Setting and Participants

The UK Biobank contains health and genetic variables for 502,649 individuals aged 40 to 69 living in the United Kingdom (UK) from 2006 to 2010. The National Health Service Register randomly sent around 9.2 million invitation letters to potential participants with a place of residency within a 25-mile radius of a UK Biobank collection centre. These collection centres were located in 22 cities across the UK, and the response rate was 5.5%. Participants went to an assessment centre where they completed an automated questionnaire, a verbal interview, and several cognitive tests, and where physical measurements and biological samples were taken. The inclusion criteria for our study were individuals born from December 1952 to July 1956, with non-missing place of birth and matched prospective hospital records.

UK Biobank received ethical approvals from the North West Multi-Centre Research Ethics Committee, the Community Health Index Advisory Group, the Patient Information Advisory Group, and the National Health Service National Research Ethics Service.

### Hospitalizations by Cause

The outcomes were generated using hospitalization data from 1997 to 2020 obtained by the Access Request Service (DARS), managed by NHS Digital. The datasets are called Hospital Episode Statistics (HES). The HES comes from routine provider data to the NHS Digital for payment and commissioning healthcare in England and is linked to 87% of the UK Biobank sample.

To identify the conditions most likely affected by in utero GLS exposure, we reviewed recent studies that estimated associations between in utero pollution exposure and health outcomes (Appendix 1). The health conditions that have received the attention of previous research are respiratory, circulatory, neoplasms, mental health, and nervous system disorders. Therefore, we concentrate on these causes of hospitalization and create five binary indicators representing at least one hospitalization between 1997 and 2020, measured by the International Classification of Disease version 10 (ICD-10 codes).

### Place-Date of Birth and Individual-Level Covariates

Biobank respondents’ year and place of birth were obtained through self-reported information. This information was used to determine whether individuals were born during the GLS (December 1952 until August 1953) or afterwards (September 1953 until July 1956), and whether they were born in London or the rest of England. Since the implementation of the Clean Air Act of 1956 had a significant impact on individuals’ health outcomes [22], we limited our sample to those born before August 1956.

Based on previous scientific literature [9], the study adjusted for demographic covariates (age at survey, sex). These variables are exogenous to the event of the GLS. The main results were also stratified by childhood socioeconomic status (SES), measured as whether an individual was born in a borough or county where the average years of completed schooling (for those born between August 1956 and December 1959) are above or below the median (alternatively below the 25th percentile or above the 75th percentile).

### Potential Bias

Our estimates may be subject to various biases. First, a potential survival bias could result from the GLS causing stillbirth and death. Second, the UK Biobank does not represent the UK population because it relies on volunteers, who tend to have higher SES [23]. It is a plausible assumption that individuals who survived the GLS and those with higher adulthood SES may exhibit better overall health than their counterparts. As a result, our sample is likely skewed towards individuals with better health and so our estimates may underestimate adverse health effects. Third, extreme events and natural disasters can influence fertility decisions [24–26], and so the GLS could have caused short-term demographic changes. In a robustness check, we exclude individuals conceived 6 months and 1 year after the GLS.

### Statistical Analysis

Difference-in-difference (DiD) regressions were estimated using ordinary least squares. The standard errors were clustered at the postcode area level with 113 clusters (21 in the treatment group and 92 in the control group), with a mean of 321 individuals per cluster. This regression approach is represented by:

$${Health}_i^h={\beta}_0+{\beta}_1{London}_i\ast {inuteroGLS}_{\textrm{i}}+{\beta}_2{London}_{\textrm{i}}+{\beta}_3{birthdate}_{\textrm{i}}+{\beta}_4{surveyyear}_{\textrm{i}}+{\beta}_5{surveymonth}_{\textrm{i}}+{\beta}_6X{\prime}_{\textrm{i}}+{\varepsilon}_{\textrm{i}}$$

The outcome variable ${health}_i^h$indicates the probability of being hospitalized from 1997 to 2020 with main cause of hospitalization *h* (respiratory, circulatory, neoplasms, mental health, and nervous system conditions). The interaction term London*inuteroGLS represents being born in London and being in utero at the time of the GLS, as opposed to being born outside of London and/or being born 1 to 35 months after the GLS. The regression also included one covariate representing being born in London (*London*_i_), 42 covariates representing year-month of birth (*birthdate*_i_), four covariates representing year of Biobank survey (*surveyyear*_i_), 11 covariates representing month of Biobank survey (*surveymonth*_i_), and seven covariates representing sex and ethnicity/race (*X*_i_).

The main parameter of interest is *β*_1_, which represents the effect of in utero exposure to the GLS. It compares differences in hospitalizations among cohorts born within London to those born in the rest of England. However, there might be a potential concern that changes occurring in the rest of England may not serve as an adequate counterfactual. To explore this issue, we considered alternative control groups including the six biggest cities in England (Birmingham, Liverpool, Manchester, Sheffield, Leeds, Bristol) and the 54 cities surrounding London.

All analyses were performed using the statistical software Stata 16.

## Results

A total of 502,649 participants visited the 22 UK Biobank assessment centres from 2006 to 2010. After excluding participants that subsequently dropped out, the sample was reduced to 502,505. Of these, 58,204 were born between December 1952 and July 1956. After excluding 9846 participants with non-linked hospitalization data and 12,077 participants with missing data on place of birth, a sample of 36,281 individuals (62.33%) was available for analysis.

The hospitalization rates from 1997 to 2020 for the group exposed to the GLS equalled 20% for circulatory issues, 17% for neoplasms, 12% for respiratory diseases, 8% for nervous system conditions, and 1% for nervous system mental health conditions (Table 1). The difference between the differences (columns 1–2 compared with 3–4) was largest for the respiratory outcome at 0.03 (or 3 percentage points). The sex and ethnicity covariates (Table 1) are relatively balanced across the treatment and control groups.

**Table 1:** Descriptive statistics for the treatment and control groups

	Born in London		Born outside of London	
	In utero during GLS	Conceived after GLS	In utero during GLS	Conceived after GLS
	(1)	(2)	(3)	(4)
Outcomes				
Circulatory	0.20	0.18	0.23	0.21
	(0.40)	(0.39)	(0.42)	(0.41)
Neoplasms	0.17	0.15	0.17	0.15
	(0.38)	(0.36)	(0.38)	(0.36)
Respiratory	0.12	0.09	0.11	0.11
	(0.32)	(0.29)	(0.31)	(0.31)
Nervous system	0.08	0.09	0.10	0.09
	(0.28)	(0.28)	(0.29)	(0.29)
Mental health	0.01	0.02	0.02	0.02
	(0.12)	(0.13)	(0.14)	(0.14)
Covariates				
Male	0.45	0.43	0.44	0.43
	(0.50)	(0.50)	(0.50)	(0.50)
Age	55.63	53.87	55.31	53.53
	(0.91)	(1.18)	(0.88)	(1.18)
White	0.98	0.97	0.99	0.99
	(0.13)	(0.18)	(0.08)	(0.09)
Other	0.01	0.02	0.00	0.00
	(0.07)	(0.11)	(0.05)	(0.05)
Number of observations	1071	3572	7311	24,327

Analysis includes data for 36,281 UK Biobank participants. Variables presented in parentheses are the means and standard errors

**Fig. 1:**
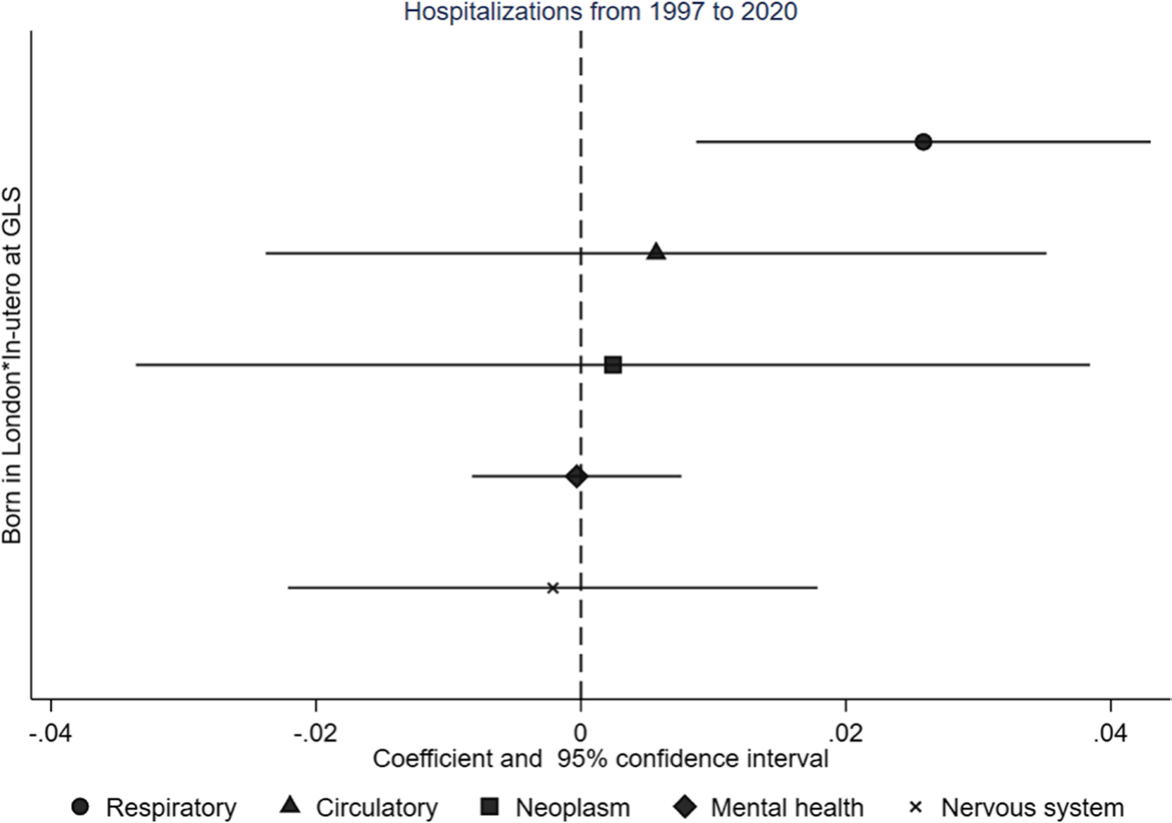
Effects of the Great London Smog on hospitalizations [Source UK Biobank. Covariates include being born in London or the rest of England, year-month birth fixed effects, sex, race, and year and month of the interview fixed effects. Coefficients and 95% confidence intervals in parentheses are presented. Standard errors clustered at the postal district level]

Figure 1 illustrates the separately estimated impacts of in utero exposure to the GLS on the five health outcomes. The results reveal a large positive effect on respiratory-related hospitalizations and small effects (close to zero) on other outcomes. Specifically, exposure to the GLS during pregnancy resulted in a 2.58 percentage point increase (95% CI 0.08, 4.30, *p* = 0.03) in the probability of hospitalization for respiratory conditions between 1997 and 2020. This effect increases the likelihood of hospitalization by 23% compared to the sample mean (11%). Importantly, these findings are robust and withstand adjustments for multiple hypotheses using Romano-Wolf standard errors [27] (Table 6).

The magnitude of the effect for respiratory health is noteworthy compared to other risk factors. For instance, the difference in hospitalizations due to respiratory conditions between individuals with and without a university degree is 2.83 percentage points, whereas the difference between individuals who have never smoked and those who have smoked is 3.45 percentage points, and the difference between males and females is 2.31 percentage points. The main causes of respiratory related hospitalizations are pneumonia (24.84%), acute lower tract respiratory infections (15.47%), asthma (7.92%), and COPD (6.28%). Estimated impacts of in utero exposure to the GLS on these four respiratory-related hospitalizations are presented in Appendix Fig. 3. The estimated effect for pneumonia is the largest; however, the confidence intervals for each outcome are large and include zero, meaning it is not possible to draw any conclusions regarding the type of respiratory illness most strongly affected by GLS exposure.

We have explored using alternative specifications to test the robustness of our results. First, we used logistic regression models rather than OLS and again found statistically significant effects only for hospitalizations due to respiratory conditions (Table 7). Second, we used two different control groups—the six most populous English cities outside of London and 54 cities and towns surrounding London—and found nearly identical estimates as those shown in Fig. 1 (Table 8). Estimates are also nearly identical when we excluded individuals conceived 6 months and 12 months after December 1952 (i.e. to control for potential short-term changes in fertility decisions) (Table 9). Finally, results are qualitatively similar when we defined outcomes based on the combination of primary and secondary causes of the hospitalization (Table 10).

Heterogeneity analyses were performed by re-estimating the regression for subsamples defined by respondent sex and SES of the boroughs or counties where they were born. The rationale for this heterogeneity analysis is that previous research has identified males as more vulnerable to in utero shocks than females, a phenomenon referred to as ‘male frailty’ [28, 29]. Additionally, stratifying by SES is important because individuals may have had different GLS exposure by SES (e.g. low SES mothers may have been working outside during their pregnancy, rather than staying indoors during the GLS). Also, individuals may have had varying means of dealing with health problems caused by the GLS due to their SES (e.g. higher SES individuals may have had better access to diagnosis and treatments).

Overall, the results do not support differential effects by sex and SES (Table 2). Focusing on respiratory health as the only statistically significant outcome, the coefficient for males is 0.02 (95% CI −0.01, 0.07) and 0.03 (95% CI 0.00, 0.042) for females. Also, when stratifying by SES, the coefficients are very similar at 0.02 (95% CI −0.01, 0.05) and 0.03 (95% CI 0.01, 0.06). *t*-tests confirm that these differences are not statistically significant. For the remaining outcomes, the results have large confidence intervals centred around zero. We have used alternative socioeconomic status (SES) definitions and found robust results (Table 11).

**Table 2:** Heterogeneity by sex and SES

Variables	(1)	(2)	(3)	(4)
	Male	Female	SES below median	SES above median
Panel A: respiratory				
Born in London*in utero at GLS	0.03	0.02	0.02	0.03
	(−0.009 to 0.069)	(0.003 to 0.042)	(−0.007 to 0.052)	(0.005 to 0.057)
Mean	0.12	0.10	0.10	0.11
Panel B: circulatory				
Born in London*in utero at GLS	0.02	−0.00	0.02	−0.01
	(−0.038 to 0.073)	(−0.035 to 0.028)	(−0.024 to 0.063)	(−0.041 to 0.028)
Mean	0.27	0.17	0.21	0.22
Panel C: neoplasms				
Born in London*in utero at GLS	0.02	−0.01	−0.02	0.02
	(−0.019 to 0.056)	(−0.059 to 0.038)	(−0.051 to 0.020)	(−0.018 to 0.061)
Mean	0.15	0.16	0.16	0.16
Panel D: mental health				
Born in London*in utero at GLS	−0.00	0.00	0.00	0.00
	(−0.015 to 0.009)	(−0.008 to 0.011)	(−0.011 to 0.012)	(−0.009 to 0.009)
Mean	0.02	0.02	0.02	0.02
Panel E: nervous system				
Born in London*in utero at GLS	0.01	−0.01	−0.01	0.01
	(−0.028 to 0.055)	(−0.047 to 0.017)	(−0.042 to 0.017)	(−0.021 to 0.034)
Mean	0.09	0.10	0.09	0.10
Observations	15,696	20,585	18,497	17,784
Baseline covariates	Yes	Yes	Yes	Yes

Source UK Biobank. Covariates include being born in London or the rest of England, year-month birth fixed effects, sex, race, and year and month of the interview fixed effects. Coefficients and 95% confidence intervals in parentheses are presented. Standard errors clustered at the postal district level

In addition, we examined whether there is variability in the effect of the GLS by whether the person was exposed in their first, second, or third gestation trimester (panel A of Table 3). We find that exposure to the GLS during the first trimester of gestation drives the overall effect on respiratory health, while exposure during subsequent trimesters has smaller effects. For the other health outcomes, small effects (close to zero) are found for each trimester of exposure.

**Table 3:** Effects of the Great London Smog at different times of exposure

	(1)	(2)	(3)	(4)	(5)
	Respiratory	Circulatory	Neoplasm	Mental	Nervous system
Panel A: effects by trimester of exposure					
Born in London*trimester 1	0.04	−0.01	−0.01	−0.01	0.00
	(0.005 to 0.075)	(−0.045 to 0.034)	(−0.056 to 0.035)	(−0.020 to 0.002)	(−0.032 to 0.035)
Born in London*trimester 2	0.02	0.00	0.01	0.00	0.01
	(−0.013 to 0.056)	(−0.044 to 0.053)	(−0.041 to 0.070)	(−0.012 to 0.016)	(−0.030 to 0.046)
Born in London*trimester 3	0.02	0.02	0.00	0.01	−0.02
	(−0.019 to 0.054)	(−0.031 to 0.065)	(−0.040 to 0.044)	(−0.011 to 0.022)	(−0.050 to 0.019)
Observations	36,281	36,281	36,281	36,281	36,281
Mean	0.11	0.21	0.15	0.02	0.09
Panel B: effects in utero and 0–24 months post birth					
Born in London*in utero	0.03	0.01	0.00	−0.00	−0.00
	(0.009 to 0.043)	(−0.024 to 0.035)	(−0.034 to 0.038)	(−0.008 to 0.008)	(−0.022 to 0.018)
Born in London*0–24 months	−0.00	0.00	0.00	0.00	0.01
	(−0.019 to 0.014)	(−0.015 to 0.019)	(−0.009 to 0.018)	(−0.004 to 0.011)	(−0.010 to 0.021)
Observations	59,166	59,166	59,166	59,166	59,166
Mean	0.11	0.22	0.16	0.02	0.09
Baseline covariates	Yes	Yes	Yes	Yes	Yes

Source UK Biobank. Covariates include being born in London or the rest of England, year-month birth fixed effects, sex, race, and year and month of the interview fixed effects. Coefficients and 95% confidence intervals in parentheses are presented. Standard errors clustered at the postal district level

Finally, in panel B of Table 3, we present estimates for a larger sample (59,166 individuals), including children exposed to GLS 0 to 24 months after birth. The results reveal small, estimated infancy effects for all health outcomes. In other words, exposure to the GLS after birth did not have identifiable long-term health impacts. Adjustments for multiple hypotheses using Romano-Wolf standard errors are found in Tables 12 and 13.

## Discussion

Air pollution is a combination of solid particles and gas in the air generated from burning fuels in industrial activities and daily consumption and naturally produced processes such as pollen and mould [30]. These substances can pass to the blood system when inhaled, and in the case of pregnant women, pollutants can reach the placenta. Black carbon has been found in the placenta of pregnant women, with its load positively correlated with residential black carbon exposure during pregnancy [31, 32]. Currently, it remains unclear how such pollutants affect the development of the foetus, but there are two documented mechanisms: (i) air pollutants reach the placenta causing inflammatory stress and molecular alterations; and (ii) air pollutants cross the placental barriers reaching the foetal system, directly affecting its developing organs [31, 32]. Both mechanisms can have a detrimental effect on the foetus’ development.

In this study, we have estimated the effect of a severe air pollution event experienced in utero on hospitalizations several decades later. It contributes to a relatively small and mostly correlational literature on the long-term health consequences of exposure to severe air pollution. The main results are that in utero exposure to the Great London Smog (GLS) increased the probability of an older-adult hospitalization due to respiratory disease by 2.58 percentage points or 23% compared to the mean. Equally important, small near-zero effects were found for other health outcomes the literature has hypothesized to be affected by pollution in utero (circulatory, neoplasms, mental health, and nervous system conditions). These results contribute to the broader literature on the ‘foetal origin hypothesis’ [13] and highlight the importance of protecting the most vulnerable individuals (those in the first months of life) during extreme air pollution to reduce long-term individual and societal costs.

The finding that in utero GLS exposure increased respiratory-related hospitalization aligns with other GLS research on respiratory health [7, 8]. However, contrary to [12], we find no effect of GLS exposure during infancy on neoplasms. Our study is the first to examine the effects of the GLS on circulatory, mental health, and nervous system conditions, which have been found previously to be correlated with (if not caused by) pollution in utero.

Heterogeneity analysis revealed that the main effect was driven by exposure to pollution during the first trimester of gestation. This can be attributed to the gradual development of the lungs, which occurs in five distinct stages: the embryonic, pseudo-glandular, canalicular, saccular, and alveolar phases [33]. Our findings indicate that the first two phases are particularly vulnerable to the effects of air pollution exposure. No significant differences were found when stratifying the results by sex and SES. This latter finding contrasts with previous research that reported a higher impact of in utero shocks on males and individuals from low SES backgrounds [4, 6].

This study has several strengths. The UK Biobank study provided a large sample of exposed individuals and relevant control groups. This meant sufficient statistical power was available to explore long-run effects (up to 69 years after the GLS) and to stratify the results by sub-groups of interest. Another strength was the ability to explore diverse, similarly-measured health outcomes through the link between the UK Biobank survey and hospitalization records. Until now, there has been no comprehensive analysis of how the GLS affected multiple health conditions in adulthood that the literature has identified as being potentially impacted by pollution exposure. This analysis provides an understanding of the relative importance of different health outcomes when considering the effect of exposure to pollution in utero.

This study is not without its limitations. The data do not allow for separate analyses by different pollutants or air pollution intensities. This reduces the external validity of the results. Although high-income countries may still experience occasional spikes of extreme air pollution, it is unlikely that they will emit high levels of certain pollutants, such as sulphur dioxide, which were prevalent during the GLS [20]. On the other hand, low-income countries frequently report levels of air pollution similar to those observed during the GLS, as burning impure fuels is more common in these regions [34].

## Figures and Tables

**Fig. 2:**
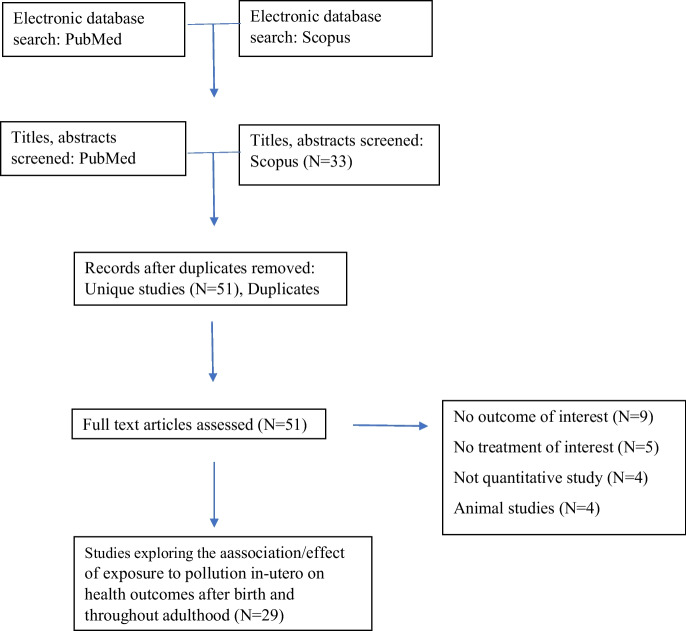
PRISMA flowchart [Source: PubMed, Scopus]

**Fig. 3:**
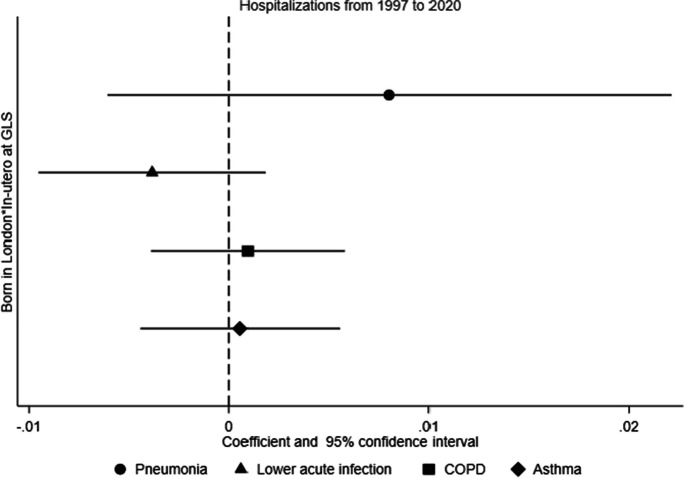
Effects of the Great London Smog on respiratory health related hospitalizations

## Data Availability

Data subject to third party restrictions.

## Appendix 1. Selecting Main Health Outcomes

### Search Strategy and Eligibility Criteria

We conducted a scoping review to identify studies that explore the association/effect of exposure to pollution in utero on health outcomes after birth and throughout adulthood. We performed an electronic literature search using PubMed and Scopus. The search strategy used the search words “air pollution AND gestation OR air pollution AND in utero OR smog AND gestation OR smog AND in utero”. Eligibility criteria were (1) at least one health outcome, (2) air pollution as main pollutant, (3) quantitative research, (4) human studies (no animal studies), and (5) published after 1st of January 2020.

The health outcomes we considered are the most prevalent and relevant causes of hospitalization (ICD-10 codes) in our UK Biobank sample. As detailed in Table 4, these include digestive, musculoskeletal, genitourinary, circulatory, blood, neoplasm, contact, respiratory, skin, eye, injuries, nervous system, parasitic, endocrine, mental health, and ear conditions. Congenital, pregnancy, external, special, and perinatal conditions are very rare in our sample and are not very relevant in our context (exploring in utero effects on older adults). As such, they are excluded.

### Study Selection

One independent reviewer (N. M. B) assessed studies identified by our searches based on titles and abstracts. No quality assessment was performed since the main objective was to identify the main outcomes that have received attention of the literature so far and not to make a distinction depending on their quality.

### Search Results

Our database search yielded 2037 studies. After title and abstract screening, 51 articles were assessed for inclusion. Ultimately, we identified 29 papers of which two are dedicated to circulatory health, one to neoplasms, 17 to respiratory health, six to nervous system conditions, and three to mental health. Figure 2 presents a PRISMA flow diagram with each part of the process, and Table 5 summarizes the number of articles found for each outcome.

Although the quality, methodology, and results of these papers varied considerably, our review shows that respiratory, circulatory, neoplastic, mental health, and nervous system conditions have received the most attention in the literature. Thus, we chose to base our analysis on these outcomes.

**Table 4:** Frequency of primary reasons for hospitalization, as classified by the ICD-10 code system, from the period of 1997 to 2020

ICD-10 code	(1)	(2)
	Average	Standard deviation
Digestive	0.47	0.50
Musculoskeletal	0.32	0.46
Genitourinary	0.28	0.45
Circulatory	0.21	0.41
Blood	0.21	0.41
Neoplasm	0.16	0.36
Contact	0.16	0.37
Respiratory	0.11	0.31
Skin	0.11	0.32
Eye	0.11	0.31
Injuries	0.13	0.34
Nervous system	0.09	0.29
Parasitic	0.05	0.23
Endocrine	0.03	0.18
Mental health	0.02	0.14
Ear	0.02	0.14
Congenital	0.01	0.09
Pregnancy	0.01	0.09
External	0.00	0.00
Special	0.00	0.04
Perinatal	0.00	0.01

Source: UK Biobank

**Table 5:** Literature on most prevalent ICD-10 hospitalization conditions

ICD-10 conditions	Paper
Digestive	
Musculoskeletal	
Genitourinary	
Circulatory	[35, 36]
Blood	
Neoplasm	[37]
Contact	
Respiratory	[38–54]
Skin	
Eye	
Injuries	
Nervous system	[55–60]
Parasitic	
Endocrine	
Mental	[61–63]
Ear	

Source: PubMed, Scopus

## Appendix 2. Additional Results

**Table 6:** Romano-Wolf multiple hypothesis testing Fig. 1

	Model *p*-value	Resample *p*-value	Romano-Wolf *p*-value
Respiratory			
Born in London*in utero at GLS	0.0035	0.0099	0.0099
Circulatory			
Born in London*in utero at GLS	0.703	0.5644	0.9802
Neoplasms			
Born in London*in utero at GLS	0.8944	0.9406	1
Mental health			
Born in London*in utero at GLS	0.9369	0.8614	1
Nervous system			
Born in London*in utero at GLS	0.833	0.7327	1

Source UK Biobank. Covariates include being born in London or the rest of England, year-month birth fixed effects, sex, race, and year and month of the interview fixed effects. Coefficients and 95% confidence intervals in parentheses are presented. Standard errors clustered at the postal district level

**Table 7:** Effects of the Great London Smog on hospitalizations estimated with logit

Variables	(1)	(2)	(3)	(4)	(5)
	Respiratory	Respiratory	Respiratory	Respiratory	Respiratory
Born in London*in utero at GLS	0.28	0.05	0.02	−0.04	−0.03
	(0.107 to 0.449)	(−0.139 to 0.231)	(−0.236 to 0.274)	(−0.537 to 0.448)	(−0.275 to 0.223)
Observations	36,281	36,281	36,281	36,281	36,281
Baseline covariates					

Source UK Biobank. Covariates include being born in London or the rest of England, year-month birth fixed effects, sex, race, and year and month of the interview fixed effects.

Coefficients and 95% confidence intervals in parentheses are presented. Standard errors clustered at the postal district level

**Table 8:** Effects of the Great London Smog (GLS) on hospitalizations considering different control groups

Variables	(1)	(2)	(3)	(4)	(5)
	Respiratory	Circulatory	Neoplasm	Mental	Nervous system
Panel A: controls six biggest cities England					
Born in London*in utero at GLS	0.03	0.00	0.01	0.00	−0.00
	(0.006 to 0.051)	(−0.035 to 0.037)	(−0.027 to 0.054)	(−0.009 to 0.012)	(−0.033 to 0.025)
Observations	12,125	12,125	12,125	12,125	12,125
Panel B: controls 54 cities surrounding London					
Born in London*In-utero at GLS	0.03	0.00	−0.03	−0.00	−0.00
	(−0.000 to 0.068)	(−0.041 to 0.043)	(−0.093 to 0.034)	(−0.019 to 0.016)	(−0.050 to 0.044)
Observations	6111	6111	6111	6111	6111

Source UK Biobank. Covariates include being born in London or the rest of England, year-month birth fixed effects, sex, race, and year and month of the interview fixed effects.

Coefficients and 95% confidence intervals in parentheses are presented. Standard errors clustered at the postal district level

**Table 9:** Effects of the Great London Smog (GLS) on hospitalizations excluding 6 months and 1 year after the GLS

Variables	(1)	(2)	(3)	(4)	(5)
	Respiratory	Circulatory	Neoplasm	Mental	Nervous system
Panel A: exclude 6 months after GLS					
Born in London*in utero at GLS	0.03	0.00	0.00	0.00	−0.00
	(0.010 to 0.046)	(−0.025 to 0.030)	(−0.034 to 0.040)	(−0.008 to 0.008)	(−0.023 to 0.016)
Observations	31,302	31,302	31,302	31,302	31,302
Panel B: exclude 12 months after GLS					
Born in London*in utero at GLS	0.02	0.00	0.00	0.00	−0.00
	(0.004 to 0.044)	(−0.026 to 0.031)	(−0.034 to 0.040)	(−0.008 to 0.010)	(−0.021 to 0.017)
Observations	25,926	25,926	25,926	25,926	25,926
Baseline covariates	Yes	Yes	Yes	Yes	Yes

Source UK Biobank. Covariates include being born in London or the rest of England, year-month birth fixed effects, sex, race, and year and month of the interview fixed effects.

Coefficients and 95% confidence intervals in parentheses are presented. Standard errors clustered at the postal district level

**Table 10:** Primary and secondary causes of hospitalizations

Variables	(1)	(2)	(3)	(4)	(5)
	Respiratory	Circulatory	Neoplasm	Mental	Nervous system
Born in London*in utero at GLS	0.03	0.01	-0.00	0.02*	0.01
	(0.009 to 0.043)	(−0.027 to 0.051)	(−0.039 to 0.032)	(−0.004 to 0.051)	(−0.012 to 0.033)
Observations	36,281	36,281	36,281	36,281	36,281

Source UK Biobank. Covariates include being born in London or the rest of England, year-month birth fixed effects, sex, race, and year and month of the interview fixed effects.

Coefficients and 95% confidence intervals in parentheses are presented. Standard errors clustered at the postal district level

**Table 11:** Other definitions of SES

Variables	(1)	(2)
	Lowest 25th percentile	Highest 75th percentile
Panel A: respiratory		
Born in London*in utero at GLS	0.03	0.02
	(−0.014 to 0.079)	(−0.019 to 0.064)
Panel B: circulatory		
Born in London*in utero at GLS	−0.30	−2.34
	(−6.484 to 5.879)	(−10.085 to 5.411)
Panel C: neoplasms		
Born in London*in utero at GLS	−1.02	−0.50
	(−5.442 to 3.402)	(−5.425 to 4.415)
Panel D: mental health		
Born in London*in utero at GLS	−0.20	1.03
	(−4.590 to 4.183)	(−5.961 to 8.027)
Panel E: nervous system		
Born in London*in utero at GLS	4.29	−1.80
	(−1.424 to 10.003)	(−5.749 to 2.153)
Observations	10,414	8608

Source UK Biobank. Covariates include being born in London or the rest of England, year-month birth fixed effects, sex, race, and year and month of the interview fixed effects.

Coefficients and 95% confidence intervals in parentheses are presented. Standard errors clustered at the postal district level

**Table 12:** Romano-Wolf multiple hypothesis testing Table 3, panel A

	Model *p*-value	Resample *p*-value	Romano-Wolf *p*-value
Respiratory			
Born in London*in utero at GLS	0.0033	0.0099	0.0099
Born in London*0–24 months at GLS	0.7631	0.6139	1
Circulatory			
Born in London*in utero at GLS	0.7085	0.6139	1
Born in London*0–24 months at GLS	0.8071	0.7624	1
Neoplasms			
Born in London*inutero at GLS	0.9008	0.8119	1
Born in London*0–24 months at GLS	0.4885	0.4257	0.9703
Mental health			
Born in London*in utero at GLS	0.9336	0.9703	1
Born in London*0–24 months at GLS	0.4182	0.2673	0.9604
Nervous system			
Born in London*in utero at GLS	0.8704	0.8416	1
Born in London*0–24 months at GLS	0.488	0.3564	0.9703

Source UK Biobank. Covariates include being born in London or the rest of England, year-month birth fixed effects, sex, race, and year and month of the interview fixed effects.

Coefficients and 95% confidence intervals in parentheses are presented. Standard errors clustered at the postal district level

**Table 13:** Romano-Wolf multiple hypothesis testing Table 3, panel B

	Model *p*-value	Resample *p*-value	Romano-Wolf *p*-value
Respiratory			
Born in London*trim 1	0.0275	0.0099	0.1089
Born in London*trim 2	0.2189	0.1287	0.7723
Born in London*trim 3	0.3521	0.2475	0.9703
Circulatory			
Born in London*trim 1	0.7918	0.703	1
Born in London*trim 2	0.7712	0.6832	1
Born in London*trim 3	0.4725	0.3465	0.9901
Neoplasms			
Born in London*trim 1	0.6581	0.5644	1
Born in London*trim 2	0.6147	0.505	1
Born in London*trim 3	0.9236	0.9406	1
Mental health			
Born in London*trim 1	0.0902	0.0396	0.3366
Born in London*trim 2	0.7594	0.6832	1
Born in London*trim 3	0.5111	0.3564	0.9901
Nervous system			
Born in London*trim 1	0.9312	0.8911	1
Born in London*trim 2	0.6955	0.604	1
Born in London*trim 3	0.3817	0.3168	0.9802

Source UK Biobank. Covariates include being born in London or the rest of England, year-month birth fixed effects, sex, race, and year and month of the interview fixed effects.

Coefficients and 95% confidence intervals in parentheses are presented. Standard errors clustered at the postal district level
